# Pandemic policy assessment by artificial intelligence

**DOI:** 10.1038/s41598-022-17892-8

**Published:** 2022-08-16

**Authors:** Sirui Song, Xue Liu, Yong Li, Yang Yu

**Affiliations:** 1 McGill University, Montreal, Canada; 2 Tsinghua University, Beijing, China; 3 Shanghai Qi Zhi Institute, Shanghai, China

**Keywords:** Computer science, Software

## Abstract

Mobility-control policy is a controversial nonpharmacological approach to pandemic control due to its restriction on people’s liberty and economic impacts. Due to the computational complexity of mobility control, it is challenging to assess or compare alternative policies. Here, we develop a pandemic policy assessment system that employs artificial intelligence (AI) to evaluate and analyze mobility-control policies. The system includes three components: (1) a general simulation framework that models different policies to comparable network-flow control problems; (2) a reinforcement-learning (RL) oracle to explore the upper-bound execution results of policies; and (3) comprehensive protocols for converting the RL results to policy-assessment measures, including execution complexity, effectiveness, cost and benefit, and risk. We applied the system to real-world metropolitan data and evaluated three popular policies: city lockdown, community quarantine, and route management. For each policy, we generated mobility-pandemic trade-off frontiers. The results manifest that the smartest policies, such as route management, have high execution complexity but limited additional gain from mobility retention. In contrast, a moderate-level intelligent policy such as community quarantine has acceptable execution complexity but can effectively suppress infections and largely mitigate mobility interventions. The frontiers also show one or two turning points, reflecting the safe threshold of mobility retention when considering policy-execution errors. In addition, we simulated different policy environments and found inspirations for the current policy debates on the zero-COVID policy, vaccination policy, and relaxing restrictions.

## Introduction

The COVID-19 pandemic has spread for almost 3 years and has killed numerous people. Nonpharmacological policies that cut off the spread of the virus through the network formed by human behaviors play a crucial role in pandemic control. The current nonpharmacological approaches include two types: individual behavior management and population mobility control. The former, such as social distancing^1^ and the contact-tracing policy^2^, aims to avoid the spread of the virus between individual contacts from a micro perspective. Population mobility control, such as the city lockdown policy and the community lockdown policy^3,4^, aims to reduce the probability of the spread of the virus across the whole society from a macro perspective. Individual behavior management is difficult to enforce and raises the concern of privacy protection^5^. In particular, it is a challenge to identify all risky individuals when the pandemic has spread widely and many asymptomatic infected people exist^6–8^. Thus, population mobility control is widely adopted by governments as an enforceable pandemic-control approach. For instance, at the initial stage of the pandemic, China adopted strict lockdown policies and successfully stopped the virus from rapidly spreading^9^.

Population mobility control yields significant economic losses and thus currently confronts strong political opposition^10–14^. Population mobility control prevents virus spread by restricting or cutting off the daily-commute network that connects different regions in a city^15–17^. For instance, the city lockdown policy uniformly restricts mobility from every urban region; the community lockdown policy cuts off the mobility originating from risky regions that have high infection rates^3,4^. However, cutting off interregional mobility simultaneously prevents healthy people who are the majority in the population from economic activities and thus brings large social costs and economic loss^10–14^. For governments, it is necessary and challenging to properly determine the extent of mobility restriction to balance the social/economic cost and pandemic suppression.

Systematic policy assessment plays a key role in the modern scientific decision-making process of governments. Analytically examining the costs, benefits, execution complexity, and risks of alternative policies not only supports but also legitimates governmental decisions^18,19^. In current real-world practices, population mobility-control policies are heuristically designed and implemented according to expert experience. Heuristic mobility-control policies expose society to the risk of experts’ or the government’s wrong decisions. The US failed to give strict control policies in the initial 2 months of the pandemic and caused catastrophic pandemic spread^20^. The lack of an analytical framework also causes difficulties in executing or legitimizing a mobility-control policy. For instance, it is currently difficult to judge whether the timing of a city’s lockdown and reopening is appropriate^21^. Furthermore, the lack of an analytical framework makes it difficult for governments to dynamically adjust their control policies to adapt to the time-varying pandemic progress or to fit different available health care capacities^22^. It is urgent to develop an analytical framework for systematically assessing population mobility-control policies.

In recent years, big technology has been widely used to assist decision-making in smart cities^23–26^. Particularly in the COVID period, -driven methods are used for pandemic simulation^17,27,28^ and pandemic risk estimation^29,30^. Previous work also used reinforcement learning (RL) to determine optimal pandemic-control policies in certain conditions^14,31,32^. However, those RL policies are rarely used in the real world. A single optimal policy may not be applicable to different socioeconomic environments because different countries, regions, or cities have different health care resources and economic vulnerabilities. It’s more informative to provide analysis and comparison of alternative policies in different conditions to governments, rather than a single optimal policy. In addition, public policy requires comprehensive decision factors rather than pure RL results^18,19^. There is still a lack of a systematic assessment framework to evaluate and compare the execution complexity, costs, benefits, and risks of different pandemic policies.

**Figure 1:**
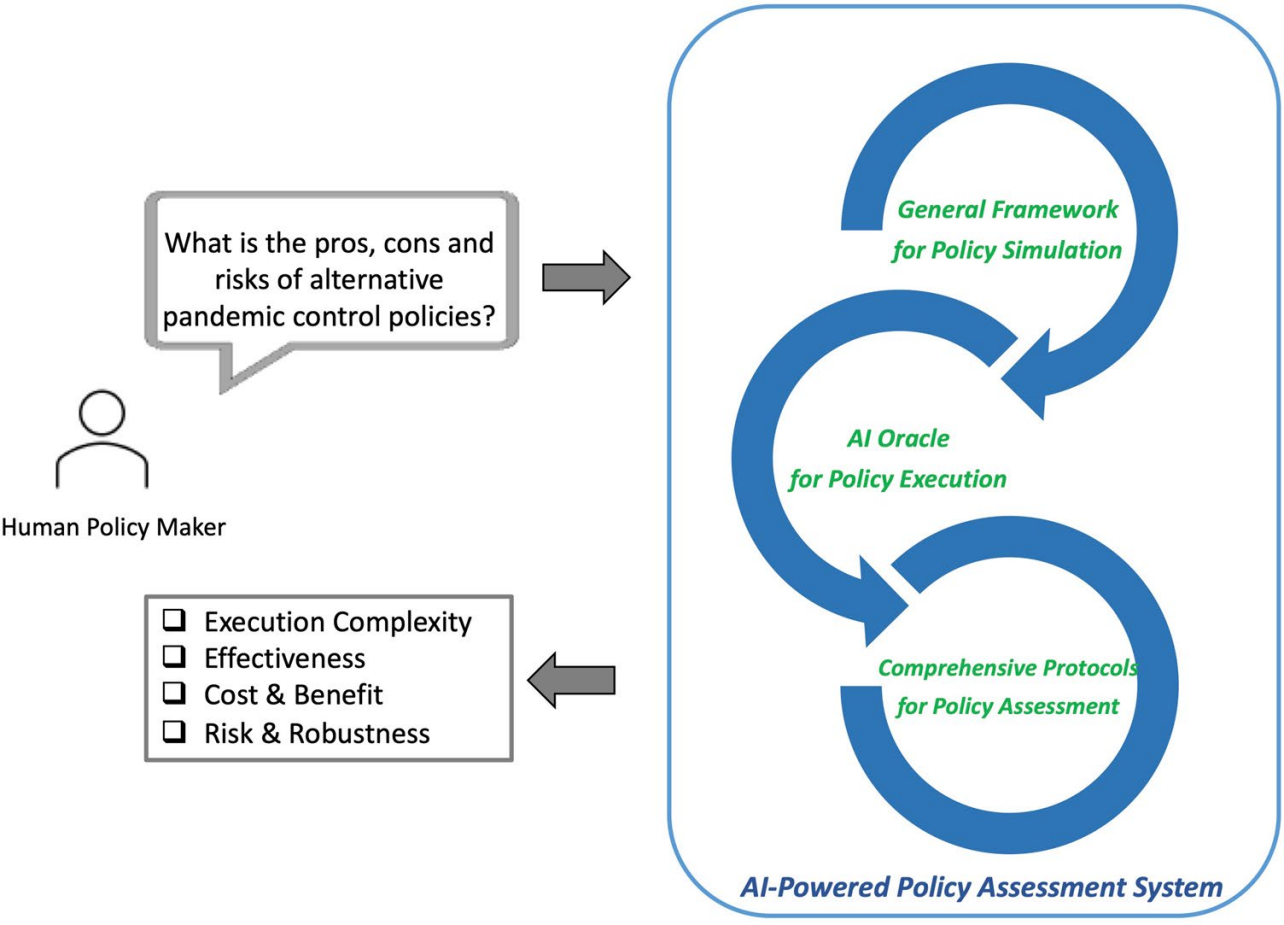
Workflow and components of the proposed AI-powered policy assessment system.

In this paper, we propose a pandemic policy assessment system that employs AI to support policy analysis (Fig. 1). The proposed system includes three components:

1. A general mobility-control framework for pandemic simulation. This framework can translate alternative real-world mobility-control policies to mathematics models and simulate how the virus spreads via mobility.
2. An adaptive AI oracle for exploring the upper bound of pandemic-control results. The oracle utilizes multiple RL models to execute each policy, and the control results are regarded as the potential upper bound of the policy.
3. Comprehensive policy assessment protocols. The protocols translate the RL-controlled results into metrics that support policy assessment in the discipline of public management, including policy-execution complexity, policy effectiveness, costs and benefits, and risks.

Using the proposed AI-Powered Policy Assessment System, we perform case studies on a real-world urban mobility network. Various kinds of mobility-control policies are assessed and compared in different environments. We further discuss the insights of policy assessment results to real-world policy-making. To conclude, the contributions of this paper are threefold:

- We propose a novel policy assessment system that can assess and compare the execution complexity, costs, benefits, and risks of a pandemic policy. The system includes three components: a general mobility-control framework, an adaptive AI oracle, and comprehensive policy assessment protocols.
- We applied the proposed system to a real-world mobility dataset collected in Beijing and analyzed three types of mobility-control policies: city lockdown, community quarantine, and route management. For each policy, we generated frontiers presenting the cost-benefit relationship between mobility retention and pandemic suppression.
- We simulated different policy environments and found inspirations for the current policy debates on the zero-COVID policy, vaccination policy, and relaxing restrictions.

*Paper organization*: In “AI-powered policy assessment system” section, we introduce the three components of the proposed policy assessment system. Then, in “Case study” section, taking Beijing as an example case, we use the proposed system to study the performance of different pandemic policies under different conditions. In “Conclusion and discussion” section, we discuss and conclude the paper. In “Methodology” section, we provide methodology details.

*Term disambiguation*: In this paper, “policy” refers to a course or principle of action adopted or proposed by a government, e.g., city lockdown. The “policy” term in reinforcement learning (which means how to perform or execute a government policy, e.g., when and how to lock down the city) is not used.

## AI-powered policy assessment system

In this section, we introduce the three components of the proposed pandemic policy assessment system: a mobility-control framework, an AI oracle, and policy assessment protocols.

### General mobility-control framework for pandemic-control simulation

In the real world, mobility-control policies have been widely adopted for pandemic prevention. These mobility-control policies can be mainly divided into city lockdown, community quarantine, and route management. Cities that are under severe pandemic status, such as Wuhan (Jan. 2020)^3,4^ and London (Mar. 2020)^33^, implement the city lockdown policy, which indiscriminately forbids all mobility in the city. For other Chinese cities in late 2020 and 2021, as the pandemic spread came under control, the government usually selectively restricted mobility for a few risky communities^34^, which is called the community quarantine policy. Some EU countries, such as Italy, allow necessary mobility during a lockdown but require residents to claim their mobility purpose^35^, which can be viewed as a more customized control for the mobility flow on each route between urban regions. For a fair assessment and comparison of these policies, it is necessary to design a general framework that can simulate different kinds of policies.

**Figure 2:**
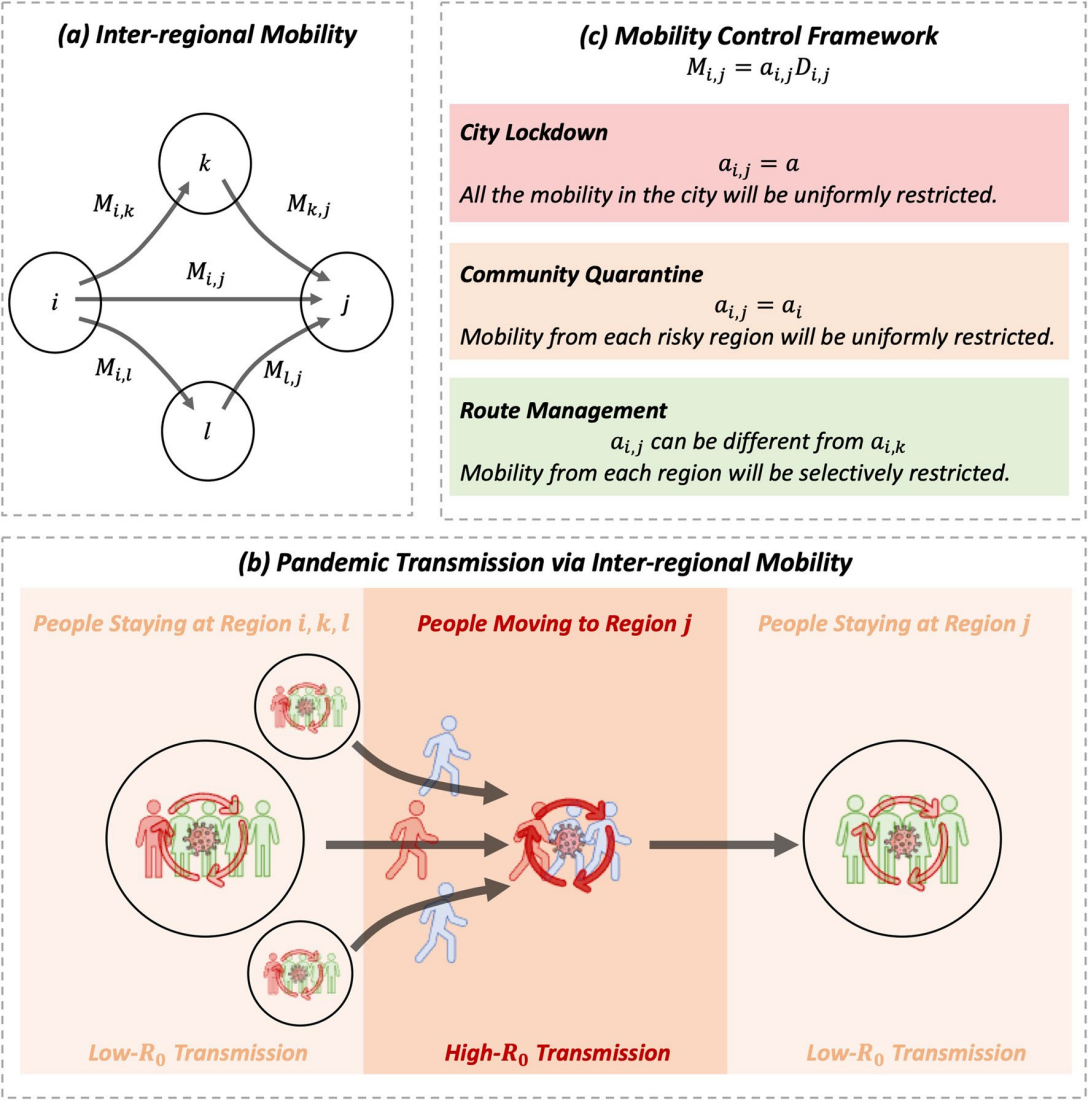
(**a**) Urban interregional mobility. (**b**) The modeling for pandemic transmission via mobility. (**c**) The modeling of interregional mobility control.

All the above mobility-control policies can be modeled as a network-flow control problem on a mobility graph^31^: in the period of $\tau$, the government restricts the mobility flow between two urban regions according to the observable infection statistics and the collection/prediction of residents’ mobility demand $D^\tau$. Assuming the expected number of residents who demand to move from region *i* to region *j* is $D_{i,j}^\tau$, the government will determine $a_{i,j}^\tau$, a certain ratio of the allowed demand. As shown in Fig. 2a, the actual allowed mobility $M_{i,j}^\tau = a_{i,j}^\tau D_{i,j}^\tau$.

The reason for governments to conduct such mobility-control policies is that the spread of a pandemic is closely related to human mobility^15,16^. As illustrated in Fig. 2b, more mobility will lead to more human contacts and further lead to more infections. Thus, controlling mobility is the key to suppressing infections.

Different governments may adopt different control resolutions according to their policy-execution ability. We show the modeling for the city lockdown policy, community quarantine policy, and route management policy in Fig. 2c. The city lockdown policy constraints $a_{i,j}^\tau = a_i^\tau$. The community quarantine policy constraints $a_{i,j}^\tau = a_i^\tau$. The most precise control is the route management policy, which customizes a mobility restriction for each route between two urban regions.

### Adaptive AI oracle for exploring upper bound of pandemic control

Exploring the ideal control results of different policies is challenging for human experts. Executing a policy well and obtaining upper-bound control results require accurately estimating the infection risks induced by mobility and the economic costs of restricting mobility and then properly determining whether, when, and where a city has to be locked down and reopened. Urban mobility is highly complex and dynamic, making it difficult to precisely estimate the risk of mobility between different pairs of regions in different time periods. Furthermore, the problem of when, where, and how to control defines a large action space, making it extremely challenging for humans to explore. As a result, current human-designed pandemic-control policies either uniformly restrict a large number of regions together or maintain restrictions for a long period. The potential of different policies remains unexplored.

Recently, reinforcement learning (RL) has been developed for solving complex control problems^36,37^. RL has also been explored in designing pandemic prevention policies^32,38,39^. However, previous works have mainly focused on making specific policies rather than systematically assessing and comparing alternative policies. In this paper, based on our proposed general mobility-control framework, we develop an RL framework^31^ that is general enough to execute different kinds of pandemic policies. Our RL system, named DURLECA, is enhanced by a specially designed GNN. DURLECA views urban mobility as a dynamic graph and utilizes the GNN to estimate the virus transmission risk induced by mobility. Then, the RL agent of DURLECA periodically determines the extent of mobility restrictions for all interregional mobility pairs according to their estimated risks.

A mobility-control policy brings economic costs and social costs^10–14^ because most mobility demands are also the foundation of people’s daily activities. During the “new norm” of the COVID-19 pandemic, an ideal pandemic prevention policy is expected to retain economic activities as much as possible while keeping the pandemic under control with constraints in health care resources, policy-execution abilities, etc. Thus, we formulate pandemic control as a dual-objective sequential control problem and train DURLECA^31^ upon it. Denoting a control policy as $\pi$, the targeted policy $\pi ^*$ is calculated in Eq. (1):

1 $$\begin{aligned}&\pi ^* = \mathrm {arg}\,\underset{\pi }{\mathrm {max}} \sum _{t=\tau }^T {\mathcal {O}}(M^{t}, E^{t+1}|a^t \sim \pi ), , \end{aligned}$$

where is the dual-objective function. ${\mathcal {O}}$ satisfies $\partial ^2 {\mathcal {O}}/\partial M_p \partial E_p < 0$ because of the trade-off nature between infection suppression and mobility retention.

The dual objectives reflect the balance between restricting mobility and suppressing the pandemic, both of which can be further modeled according to real-world policy environments. For example, people’s endurance for continuous and long-term lockdown can be modeled as a booming cost for continuously restricting one region; the city’s limited health care resources can be modeled as a skyrocketing cost if the demand for hospitalizations exceeds the city’s health care capacity. In the Methodology section, we further introduce the design of the model and the objective function.

Policies found by RL may be complex and impose high requirements on real-world policy execution. However, the results of those policies can be viewed as an indicator to evaluate the potential of real-world policies, i.e., the best control result people can obtain in the real world. For every policy we want to evaluate, we train multiple RL models under different conditions. The upper bound of those models is recorded and analyzed. With additional execution complexity analysis (introduced later), the results of different RL-based policies can serve as an informative reference for policy-makers to assess and compare policies.

### Comprehensive policy assessment protocols

In this section, we introduce four kinds of policy assessment protocols. The first protocol analyzes the execution complexity of a policy. The other three protocols translate a policy’s control results to analyzable and comparable metrics.

#### Policy-execution complexity and requirements

The complexity of a policy poses requirements on a government’s execution ability. A government with high execution ability can adopt policies that require more control preciseness and dynamics. In the real world, governments in different countries have different execution abilities. For each kind of policy, we have to know how hard it is for governments to perform it well.

We evaluate a policy’s execution complexity by its temporal and spatial control resolution and pattern. For example, community quarantine is complex: it needs $N_c$ (the number of communities) actions per control period; in addition, ideally performing community quarantine requires a community-coordination pattern; we conclude that such a policy asks the municipal government to have flexible control over communities and thus is not suitable for governments with low execution ability.

#### Effectiveness of retaining mobility and flattening hospitalizations

The most critical metrics for evaluating a pandemic-control policy are how it retains mobility and how it flattens hospitalizations or infections. In particular, the peak number of hospitalizations is a significant index for infection suppression. When peak hospitalizations exceed the capacity of a city’s health care system, the pandemic may cause catastrophic deaths and social panic. Thus, how a pandemic-control policy can lower the peak number of hospitalizations is the most prioritized index for policy effectiveness assessment. From the mobility retention side, the amount of mobility that a control policy can retain without breaking the health care threshold is the second assessment metric.

#### Cost-benefit analysis

As discussed before, a pandemic prevention policy that can suppress more infections will naturally restrict more mobility. In the “new normal” of the COVID-19 pandemic, it is of urgent demand to determine how much mobility restriction we will accept as a cost to obtain infection-suppressing gain or how much more mobility we can gain if we invest more health care resources and hold more patients. Thus, the cost-benefit relationship between mobility retention and infection suppression is the third metric for policy assessment.

For each policy to be assessed, we use the AI oracle to explore its upper-bound control results under different conditions, e.g., the minimal hospitalizations/infections when keeping 50% mobility, the minimal hospitalizations/infections when keeping 70% mobility, etc. Then, we draw two curves to present the trade-off relationship between mobility and hospitalizations/infections, namely, the mobility-hospitalization and mobility-infection frontiers.

#### Robustness and risk

In the real world, we may not guarantee the accuracy of the collected data or conduct control actions. For example, the government may not accurately collect mobility demand or the number of symptomatic patients; residents may break the mobility restriction and move from one risky region to another. These errors will cause the actual mobility in the city to be less/more than the designed mobility. In the real world, it is important for policy-makers to choose a proper mobility restriction target and ensure that a slight mobility deviation will not cause catastrophic results. This asks policy-makers to understand the safe threshold of each policy and how fast infections/hospitalizations will increase when mobility is within/beyond the safe threshold.

The mobility-hospitalization frontier and mobility-infection frontier provide an opportunity for policy-makers to understand such information. Generally, as long as the implemented policy is not at the steep part of the policy frontier, then a slight mobility change will not cause much infection increase. Thus, we say a policy’s flat curve part is robust and the steep part is sensitive to perturbations. Specifically, we use the mobility of its first turning point and its slopes at certain points as indices to analyze policy robustness.

## Case study

In this section, taking Beijing as an example case, we use the proposed system to study the performance of different pandemic policies under different conditions. The mobility-control framework is built on real-world mobility data from Beijing in Jan. 2019, covering more than 500,000 people.

### Analyze the same policy in different environments

**Figure 3:**
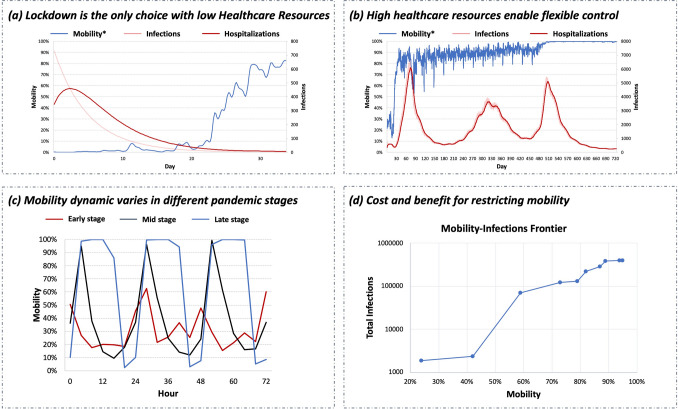
(**a**)/(**b**) The control extents and pandemic statistics of two example policies that react differently according to different given health care resources; Mobility* refers to the smoothed mobility. (**c**) Dynamic mobility restrictions of city lockdown with high health care resources in the early stage, mid-stage, and late stage of the pandemic; for each stage, we demonstrate control actions in three example days. (**d**) The trade-off relationship between mobility retention and infection suppression.

We use the proposed system to assess the city lockdown policy, as shown in Fig. 3. The policy presents different execution complexities and demonstrates diverse control results when the city’s health care resources vary.

#### Policy-execution complexity and requirements

As shown in Fig. 3a, when the city has low health care resources and cannot afford many patients, to obtain affordable infections/hospitalizations, the city needs to lock down the whole city to quickly cut off the spread of the virus until the pandemic spread slows and then gradually reopen the city. There is no space for releasing mobility or elastic control. In the real world, China’s pandemic prevention policy adopted in Wuhan satisfies such execution requirements and thus achieves good control results.

In contrast, for cities that have high health care resources (or relatively low lockdown endurance), the control result shows more mobility-retaining ability, as shown in Fig. 3b. However, to achieve such a result, policy execution needs to be more dynamic. Stringent restrictions are given for only a short period to prevent the number of infections from rapidly increasing. After short-term stringent restrictions, relatively relaxed restrictions are given to reduce the rapidly increasing lockdown cost. Infections will increase when the city relaxes restrictions. However, when the increase in infections reaches another dangerous level, the city will strictly restrict mobility again. As such, the number of infections is always under the city’s health care capacity.

The control dynamics also vary daily, and the varying pattern changes as the pandemic stages change, as shown in Fig. 3c. At the early stage, it is urgent to stop the rapidly increasing infections. Thus, a complete lockdown policy is implemented, and only little mobility is allowed. At the mid-stage of the pandemic, since the fast growth of infections has been stopped, there are chances of retaining essential day-time mobility for economic activities. However, a curfew policy is needed to reduce residents’ movements and contacts. At the late stage of the pandemic, since the population has developed immunity against the virus, more daytime mobility can be retained, and the curfew policy can be more relaxed. In summary, to achieve ideal mobility retention and always suppress infections under the city’s health care capacity, policy execution requires a long-term stringent-loose-stringent alternative and a daily curfew pattern.

#### Control results assessment

*Effectiveness*. As shown in Fig. 3a/b, the city lockdown policy has the potential to effectively retain mobility and suppress infections/hospitalizations in different situations. When the city has low health care resources, the policy keeps the peak hospitalization rate under 0.08%, which is below the hospital bed density of most countries (157 out of 179)^40^. Considering that patients with mild symptoms do not need to stay in the hospital, the actual hospitalization rate can be lower and more countries can afford it; the overall retained mobility rate is 24%. When the city has high health care resources, the policy keeps the peak hospitalization rate under 0.89%, which can be afforded by some developed countries such as South Korea and Japan; the overall retained mobility rate is 76%. There are very few stringently restricted days, and thus the economy may not be largely affected.

*Cost-benefit analysis*. We present the cost-benefit curve between mobility retention and infection suppression in Fig. 3c. As discussed before, when the city has more health care resources, it can afford more infections and retain more mobility. There are two notable turning points in the curve (mobility 42% and mobility 59%). As the retained mobility increases, the total infections and hospitalizations first grow slowly before the first turning point, forming a flat curve. Then, the first turning point comes, which means that the current control policy cannot stop the spread of the virus. After a steep increase in infections, the curve meets the second turning point and returns to a flat mode. This means that the population has developed herd immunity; increasing mobility in this case does not result in many more infections. The cost-benefit curve tells the mobility “cost” we pay to obtain infection-suppressing “gain” or the mobility “gain” we could have if we “cost” more infection-suppressing effectiveness. This can provide guidance for managing resources. For example, it will be beneficial to invest health care resources in the flat stage, where increasing the ability to hold more patients can obtain significant mobility-retaining gain.

*Robustness*. As discussed in “Comprehensive policy assessment protocols” section, the observation error or execution error in the real world may cause the actual mobility to be lower or higher than the designed mobility. The first turning point of the policy frontier curve reflects the safe mobility threshold. The curve slope within and beyond the threshold reflects how terrible the result is if there is a deviation in mobility. According to Fig. 3c, the mobility of the first turning point is 42%; the infection-increasing speed is 27 people per 1% mobility within the threshold, and 3697 people per 1% mobility beyond the threshold. For the government, it is critical to keep mobility below such a threshold when adopting a city lockdown policy.

### Analyze different policies in the same environment

**Figure 4:**
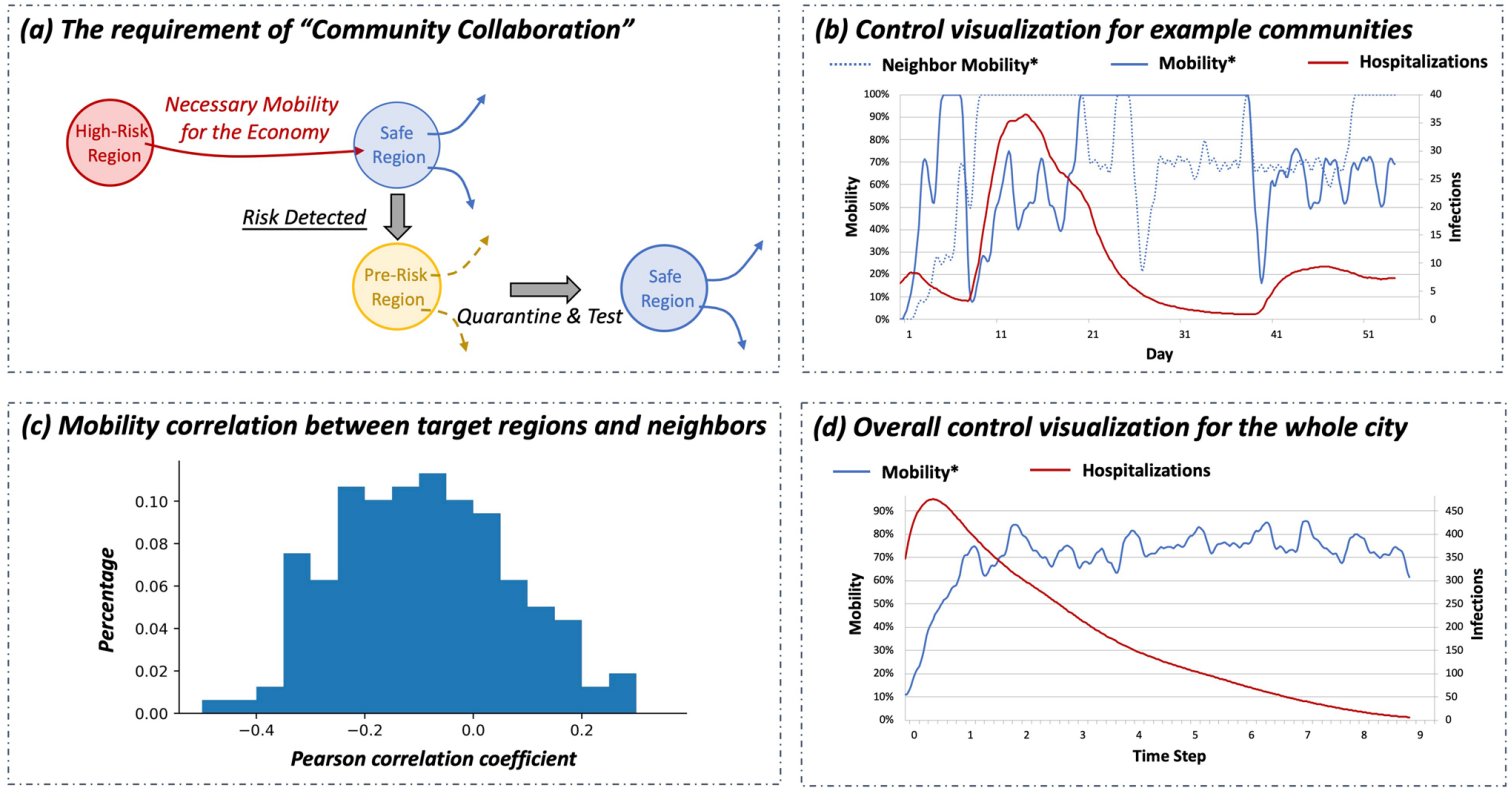
Ideally executing community quarantine and route management requires “community collaboration”. (**a**) Intuitive explanation. (**b**) The control extents and pandemic statistics of example communities; one community would accept risky mobility from another community to relieve its lockdown pressure; in the meantime, it would lock itself down to prevent further pandemic spread. (**c**) The Pearson correlation coefficient between the mobility of every region and its collaborative neighbor; for the example two communities, the coefficient is $-0.28$. (**d**) The overall control extents and pandemic statistics of the city.

**Figure 5:**
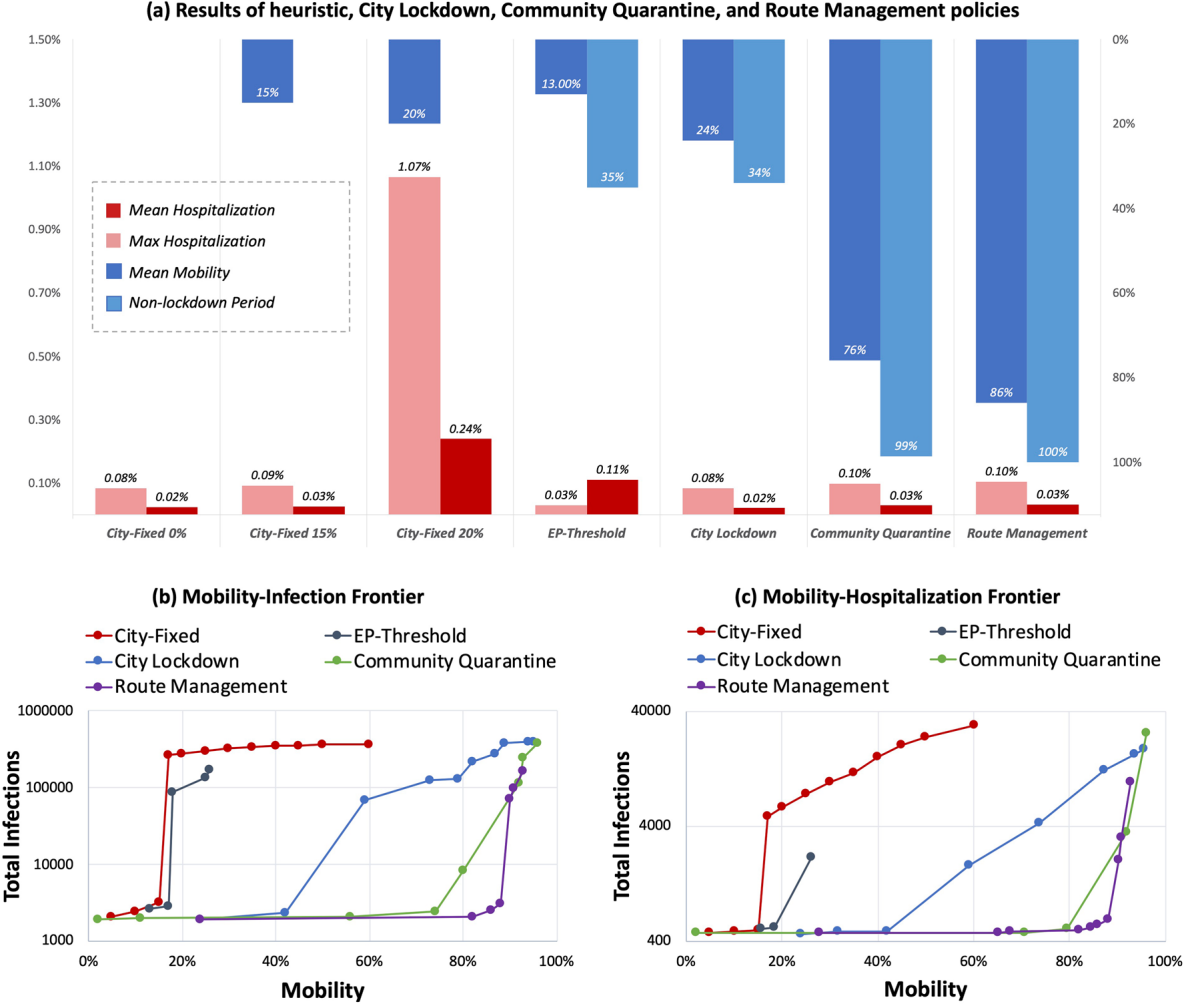
The cost-benefit relationship between mobility retention and pandemic suppression.

We use the proposed system to assess and compare different kinds of mobility-control policies, as shown in Figs. 4 and 5. We analyzed the complexity of community quarantine and route management. We also compared the results of heuristic policies (detailed in “Experiment settings” section), the city lockdown, community quarantine, and route management.

#### Policy-execution complexity and requirements

Adopting a community quarantine policy or a route management policy is more complex than implementing a city lockdown policy. A statistical comparison of the complexity of those policies is shown in Table 1.

**Table 1:** Statistical comparison of the complexity of city lockdown, community quarantine, and route management.

Policy	Action count	Daily action std
City lockdown	1	0.05
Community quarantine	323	0.38
Route management	16,285	0.36

The computation of daily action Std is detailed in “Experiment settings” section.

For a city lockdown, the government needs to determine only one action for the whole city. For community quarantine, the government needs $N_c$ diverse actions for $N_c$ communities (in our experiments, $N_c=323$). For edge control, the government needs to determine at most $N_c \times N_c$ diverse actions for routes among all communities (in the experiment mobility dataset, in total 16285 pairs of communities have valid interregional mobility).

We also find that performing a community quarantine policy or a route management policy requires communities to collaborate with each other (Fig. 4): one community serves as a “flood-relief” channel to its neighboring high-risk communities that have been continuously restricted. This is because a continuous and long-term lockdown in a community can severely hurt the economy and affect residents’ lives. Thus, some “flood-relief” communities need to tolerate mobility from high-risk communities to satisfy their urging mobility demand and alleviate their mobility-restriction burden. When opening to high-risk communities, the “flood-relief” communities need to close their own borders and stop sending out people. This prevents the “flood-relief” communities from further spreading the virus. In this way, the cost of locking down the high-risk community is significantly reduced, and the infection-spread cost increases only slightly. Without worrying about the mobility-restriction cost, high-risk communities can thus afford longer restrictions and finally lead to better pandemic suppression results.

#### Control results assessment

In Fig. 5a, we compare the effectiveness of different control policies. Furthermore, in Fig. 5b/c, we show the cost-benefit curves of those control policies. We find how increasing control preciseness or resolution can improve control results.

*Effectiveness*. Under the same environmental setting, we compare the performance of different policies in Fig. 5a. Dynamic policies (EP-threshold, city lockdown, community quarantine, route management) are much more effective than fixed policies (city-fixed 0%, city-fixed 15%, city-fixed 20%) in both mobility retention and pandemic suppression. Within dynamic policies, when keeping the same number of hospitalizations, route management retains 86% overall mobility and community quarantine retains 76% overall mobility, both of which are much more than the 24% retained mobility of city lockdown. We further discuss the benefit of improving control resolution and complexity from a temporal view and spatial view in Section 3.3.

*Cost-benefit analysis*. For city-fixed/EP-threshold/city lockdown, there are two turning points in the mobility-infection frontier. Compared with them, for community quarantine and route management, there is only one turning point, and the turning point comes very late; this means they can prevent infections/hospitalizations from rapidly increasing while retaining most of the urban mobility. Community quarantine and route management do not show a second turning point. This means that their pandemic suppression effectiveness is comparable to that of the herd immunity policy, thus leaving no more space for the later herd immunity stage.

*Robustness*. As discussed before, a policy is robust in the flat part of its frontier curve and risky in the steep part. For each policy, we use the mobility of its first turning point and its slopes at certain points to analyze policy robustness. *Intelligent policies present more robustness than heuristic policies*. The safe threshold of heuristic policies (city-fixed, EP-threshold) is below 20%. Once mobility exceeds this threshold, infections will rise sharply due to uncontrolled contacts. However, for intelligent policies (city lockdown, community quarantine, route management), the safety threshold is larger than 40% and the increase in infections/hospitalizations is much lower. These results show the benefit of identifying and restricting risky mobility. *Precise policies are more robust than coarse policies*. The safe thresholds of city lockdown, community quarantine, and route management are 42%, 80% and 88%, respectively. Within the safe threshold, route management has the slowest infection-increasing speed. When the mobility exceeds 88%, the infection-increasing speed of these three policies becomes close.

### Insights of policy assessment results

#### The benefit of improving temporal control resolution and complexity

We compare the performance of fixed control (city-fixed) and dynamic control (city lockdown, community quarantine, route management) policies to discuss how temporal control resolution affects policy effectiveness. Dynamic control greatly outperforms fixed control in both mobility retention and pandemic suppression. The reason is that temporal dynamic control enables governments to adjust the control extent according to the current pandemic risk and mobility need. For example, in the early stage of city lockdown (Fig. 3a/b), the government needs stringent control to stop the rapid increase in infections; when the infection curve has been flattened, the government can give relaxed control to satisfy socioeconomic needs. Similarly, as shown in Fig. 3d, daily control dynamics such as the curfew policy are also beneficial for mobility retention and pandemic suppression.

The above conclusion is similar to real-world practices. We conclude that it is beneficial for governments to conduct a dynamic policy instead of a fixed policy regardless of the complexity of the former.

#### The benefit of improving spatial control resolution and complexity

We further compare the performance of city lockdown, community quarantine, and route management to discuss how the spatial control resolution affects policy effectiveness. On the one hand, by improving control resolution or preciseness from city lockdown to community quarantine or route management, the control results obtain a significant benefit. This is because fine-grained control can better distinguish high-risk mobility and low-risk mobility; then, restricting the former and retaining the latter becomes possible, which finally leads to satisfactory mobility retention and pandemic suppression output. On the other hand, continuing to enhance control resolution from community quarantine to route management brings less return. This is because once a community has a high infection rate, all of its outward mobility has the same risk level; distinguishing them brings little gain.

In the real world, most governments conduct community quarantine rather than city lockdown or route management. We conclude that this choice is a balance of good pandemic-control results and acceptable policy-execution complexity.

#### Inspirations to today’s policy debate

*Zero-COVID*. The zero-COVID policy, which aims at zero new infections and resuming normal economic and social activities, is plausible in city lockdown, community quarantine, and route management. Whether to adopt a zero-COVID policy depends on the city’s health care resources. In addition, the cost of the zero-COVID policy varies according to the control resolution, as shown in Fig. 5b/c. For city lockdown, when the city has only limited health care resources and wishes to suppress infections at the flat frontier curve, complete lockdown is the only choice: stringent mobility restrictions at the beginning and gradual reopening when infections decrease significantly. The retained mobility is close to zero in the restriction stage and at most 40% in the whole pandemic period. For community quarantine, the cost is much lower, since only the infected communities and their related communities need to be restricted; at most 80% mobility can be retained. For route management, due to more precise control, at most 88% mobility can be retained when realizing the zero-COVID policy.

**Figure 6:**
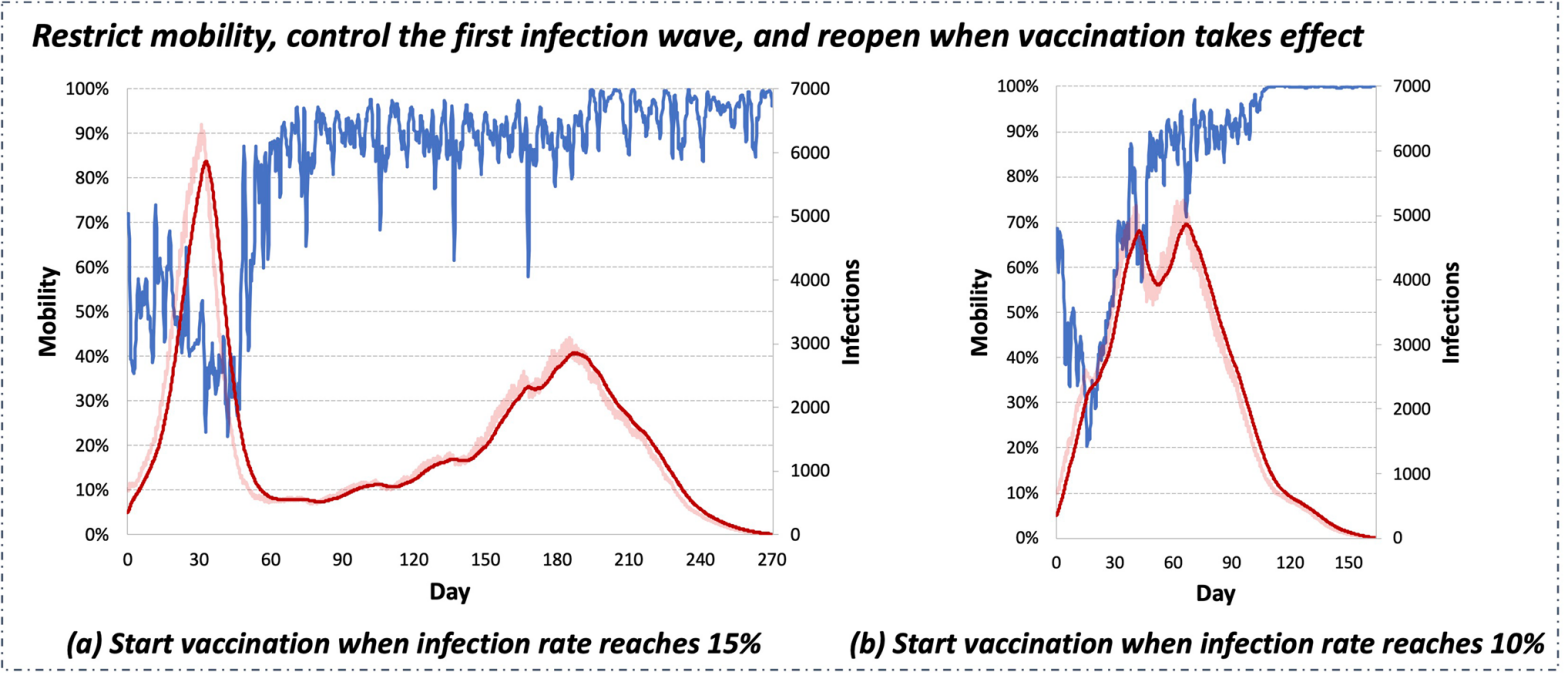
The city lockdown policy reacts differently to different vaccination settings. For both settings, the speed of vaccination is set as 0.5% of the population per day. (**a**) Start vaccination when the total infection rate reaches 15%. (**b**) Start vaccination when the total infection rate reaches 10%.

*Vaccination*. In the past year, COVID vaccines have been widely adopted all over the world to promote population immunity. In our simulation, we model the effect of vaccination as the increase in the R (recovered) population over time. Taking city lockdown as an example, we compare two different vaccination settings. As shown in Fig. 6, we find that vaccination will end the pandemic earlier. However, vaccination needs to be combined with stringent restrictions at the beginning of the pandemic to control the first infection wave and leave time for a larger population to get vaccinated.

*Restriction relaxation*. As shown in Fig. 3b, the city can relax restrictions when herd immunity is built at the end stage of the pandemic. Similarly, relaxation is observed in Fig. 6 when herd immunity is built by vaccination. Such observations also match the current relaxation trend in many countries, such as Canada and the US. Those countries adopted a flexible control policy in the past 2 years, which resulted in a large infected population. In the past year, a large portion of the population in those countries became vaccinated, which further increased their population immunity. Considering the population immunity, the flattened infection curve, and the rich health care resources, the infection cost of relaxation in those countries becomes affordable.

## Conclusion and discussion

### Conclusion

Mobility-control policy is the most common pandemic-control policy in the real world. Governments in different countries and regions adopt different kinds of policies according to their conditions. However, current policies are heuristically designed and implemented according to expert experience. There is a lack of an analytical framework enabling governments to compare different policies and determine the optimal one.

In this paper, we propose an AI-Powered Policy Assessment System to support policy analysis. The proposed system consists of (1) a general framework that can model and simulate various types of policies; (2) an AI oracle to compare alternative policies; and (3) comprehensive protocols mapping the RL results to policy assessment measures. In the AI oracle, we propose using multiple RL models for policy assessments, which suggests a novel perspective of utilizing AI in addition to using a single model to predict or search for policies.

We applied our framework to analyze different kinds of policies on real-world metropolitan data and assess policies by their execution complexity, effectiveness, costs and benefits, and robustness against risk. For each policy, we generated frontiers presenting the cost-benefit relationship between mobility retention and pandemic suppression. The assessment results manifest that the most precise policies, such as route management, have high execution complexity but limited additional gain from mobility retention. In contrast, a moderate-level intelligent policy such as community quarantine has acceptable execution complexity but can effectively suppress infections and largely mitigate mobility intervention. In addition, our assessment confirms the rationality of switching the lockdown strategy toward a curfew policy. We also find that the frontiers of different policies have different turning points, which reflect their robustness against risks. Governments need to restrict mobility below the first turning point to prevent unexpected pandemic outbreaks.

Furthermore, we simulated different policy environments to bring inspiration to the current policy debate on the zero-COVID policy, vaccination policy, and relaxing restrictions. The assessment results demonstrate the following: (1) if the city has low policy-execution ability and limited health care resources, the zero-COVID policy is the only choice; (2) it’s necessary to combine mobility control with vaccination progress; and (3) with population immunity, the flattened infection curve, and rich health care resources, it is affordable for some countries to relax mobility restrictions.

### Discussion

#### RL in the real world

We discuss the paper from a more general perspective of using RL in real-world policy design and analysis. The results of RL have randomness. We might end up with many failed training results before we find a successful result. In addition, the resulting RL model might overfit specific environmental settings and is difficult to generalize to other environments, such as other cities. These make it difficult to rely on a single RL model for policy design in the real world.

However, in this paper, we do not rely on a single RL model. Instead, multiple RL models are used to explore the upper bound of a certain policy, which can be an informative signal to help policy comparisons and support policy assessments. Such a signal can be further translated into metrics in the discipline of public management, such as the cost-benefit curve, to give policy-makers more insights. Furthermore, compared with using a single RL model to search or design a policy, the randomness and the overfitting problem of multimodel-based policy assessment will be largely reduced. From these perspectives, policy assessment is more trustworthy.

#### Generalization

The generalization of this paper can be discussed from three perspectives.

*Policy assessment workflow*. While the proposed policy assessment system is applied and studied on pandemic policies in this paper, such a system and workflow also can be applied to other policy scenarios. As long as a public policy can be simulated and people have certain optimization targets, e.g., indices to be minimized or maximized, RL can be used to explore the policy upper bound and further serve as a policy assessment tool. For example, users can adopt a transportation simulation framework and a transportation policy agent to assess daily transportation policies such as the road space rationing policy or the congestion charge policy.

*Policy assessment results*. As we discussed before, the aim of this paper is not to train a single RL model and use it to search for or design policies. Instead, multiple RL models are used to explore the upper bound of a certain policy. The possible overfitting problem or randomness problem of a single RL model will be largely reduced as we combine the results of multiple models for policy assessment. In addition, the frontier curve summarizes policy performance under different social conditions. Thus, the policy assessment results in one city are meaningful even for policy-makers in different cities.

*RL-based policy model*. The generalization of a single RL model highly depends on the input information and the utilization of information. In our experiments, the model takes mobility as input and fully utilizes it. Even though the mobility pattern changes, as we swap the mobility of different days, the previously trained RL model still works. We conclude that if the input is sufficient, the environment has no hidden parameters, and the model fully utilizes the input information, then the trained model can be generalized to other environmental settings. If the environment has hidden parameters, such as a special vaccination setting, we recommend that users retrain the RL model for the best performance.

#### Limitations and future work

The paper has two limitations. First, the current frontier curve is relatively conservative. We train multiple AI models and choose the best ones as the upper-bound results of a policy to draw the frontier curve. In this way, the chosen best models are close to the ideal results, but better models might exist if we train more times. Thus, the drawn frontier curve might be slightly more conservative than the ideal frontier curve. Our future work may consider adversarial learning methods to further approximate the ideal results. Second, the current framework considers the upper bound of a policy. In the real world, understanding the lower bound, or the worst case of a policy, is also important. Our future work may add noise or set more constraints to simulate errors in real-world policy execution and see how the control results of different policies degrade.

## Methodology

In DURLECA^31^, we develop an interregional mobility-control framework for pandemic simulation and an RL algorithm for designing route management policies. Readers can refer to the original paper^31^ or https://github.com/anyleopeace/DURLECA for technical details.

In this paper, we find that the simulation framework and RL algorithm in^31^ have potential in modeling different control resolutions and different pandemic/social environments. We first adopt the original simulation framework^31^ and improve it to be more general to model different kinds of policies. Then, we propose a crowd-intelligent design to train multiple RL models and explore the upper bounds of different policies. Finally, we simulate different environments that vary in health care resources and economic vulnerability and compare and assess policies in a controlled analytical framework.

### General simulation framework

We summarized all notations in Tables 2 and 3.

**Table 2:** The summary of pandemic-related notations.

Term/notation	Definition
Superscript $\tau$	At time step $\tau$
Subscript *i*	At region *i*
Subscript *v*	Visible pandemic state
$N_i^\tau$	The total population
$S_i^\tau$	The number of susceptible population
$I_i^\tau$	The number of infected but not identified/hospitalized population
$H_i^\tau$	The number of hospitalized population
$R_i^\tau$	The number of recovered population
$E_i^\tau$	$(S_i^\tau , I_i^\tau , H_i^\tau , R_i^\tau )$. The pandemic state
$E_{v,i}^\tau$	$(S_i^\tau +I_i^\tau , H_i^\tau , R_i^\tau )$. The visible pandemic state

**Table 3:** The summary of mobility-related notations.

Term/notation	Definition
Superscript $\tau$	At time step $\tau$
Subscript *d*	The original demand without restrictions
Subscript *p*	With restriction *p*
Subscript *i*, *j*	Region index
$M_*^\tau$	The mobility. A matrix
$M_{*,i,j}^\tau$	The OD flow from *i* to *j*. A scalar
$M_{*,i}^\tau$	$\sum _j M_{*,i,j}^\tau$. The outflow from *i*. A scalar
$\overline{M_{*,i}}$	$\frac{1}{T}\sum _\tau M_{*,i}^\tau$. The mean outflow from *i*. A scalar

Subscript $*$ can be either *d* or *p*.

*Pandemic simulation*. The simulation framework developed in^31^ defines the pandemic state of each region as $E_i^\tau =(S_i^\tau , I_i^\tau , H_i^\tau , R_i^\tau )$. The SIHR model distinguishes the *I* state (infected but not identified/hospitalized population) and the *H* state (infected and identified/hospitalized population) to simulate the real-world scenario where many asymptomatic patients are hard to identify.

The framework further models how the virus spreads via mobility^31^. The pandemic state transition is depicted by population mobility:

2 $$\begin{aligned}&E_i^{s, \tau } = E_i^{\tau } - \sum _j \frac{M_{p,i,j}^{\tau }}{N_i^{\tau }}E_i^{\tau }, \end{aligned}$$

3 $$\begin{aligned}&E_i^{m, \tau } = \sum _j \frac{M_{p,j,i}^{\tau }}{N_j^{\tau }}E_j^{\tau }, \end{aligned}$$

4 $$\begin{aligned}&{\hat{E}}_i^{\tau } = E_i^{s, \tau } + E_i^{m, \tau }, \end{aligned}$$

and virus transmission:

5 $$\begin{aligned}&S_i^{\tau +1} = {\hat{S}}_i^{\tau } - \frac{\beta _i^{s,\tau } S_i^{s,\tau } I_i^{s,\tau }}{N_i^{s,\tau }} - \frac{\beta _i^{m,\tau } S_i^{m,\tau } I_i^{m,\tau }}{N_i^{m,\tau }} - \alpha \left( N_i^{m,\tau } + N_i^{s,\tau }\right) , \end{aligned}$$

6 $$\begin{aligned}&I_i^{\tau +1} = {\hat{I}}_i^{\tau } + \frac{\beta _i^{s,\tau } S_i^{s,\tau } I_i^{s,\tau }}{N_i^{s,\tau }} + \frac{\beta _i^{m,\tau } S_i^{m,\tau } I_i^{m,\tau }}{N_i^{m,\tau }} - \gamma {\hat{I}}_i^{\tau }, \end{aligned}$$

7 $$\begin{aligned}&H_i^{\tau +1} = H_i^{\tau } + \gamma _i^\tau \hat{I_i}^{\tau } - \theta _i^\tau H_i^{\tau }, \end{aligned}$$

8 $$\begin{aligned}&R_i^{\tau +1} = \hat{R_i^{\tau }} + \theta _i^\tau H_i^{\tau } + \alpha \left( N_i^{m,\tau } + N_i^{s,\tau }\right) . \end{aligned}$$

$\{{\hat{S}}_i^{\tau },{\hat{I}}_i^{\tau },{\hat{H}}_i^{\tau },{\hat{R}}_i^{\tau }\}$ are elements of ${\hat{E}}_i^{\tau }$. $\{ \beta ^{s,\tau }_i, \beta ^{m,\tau }_i \}$ are the pandemic’s transmission rates for the staying population and the moving population, respectively. $\gamma ^\tau _i$ is the hospitalization rate, and $\theta ^\tau _i$ is the recovery rate. Particularly, to support vaccination policy discussion, we add the term $\alpha (N_i^{m,\tau } + N_i^{s,\tau })$ in this paper to reflect the effect of vaccination: in every step, a fixed portion of the population gets vaccinated and obtains immunity.

*Modeling different kinds of mobility-control policies*. In^31^, urban mobility is depicted by mobility flows from source regions to target regions; mobility control is modeled as retaining a certain percentage $a_{i,j}$ of mobility between source region *i* and target region *j*. Such modeling was originally designed for route management. However, this mobility-control framework can be used to model different kinds of policies by adjusting the control resolution. For city lockdown, we constrain $a_{i,j}^\tau = a_0^\tau$ to realize city-level control resolution. For community quarantine, we constrain $a_{i,j}^\tau = a_i^\tau$ to realize community/region-level control resolution.

### AI oracle: a crowd-intelligent design

Although RL can be used for policy-making^31^, such single-model-based policy-making is not trustable in the real world due to the randomness of RL training and the potential overfitting problem. This makes it difficult to fairly compare different kinds of policies. Furthermore, recalling that different cities may have different economic vulnerabilities or different amounts of health care resources, we need to assess a policy considering the different trade-offs between the economy and the pandemic. In this paper, we propose using multiple RL models to explore the upper bound of a policy and assess the policy based on that, referred to as crowd intelligence.

We first use the RL algorithm in^31^ for single-model training. The state of RL is defined as follows: the current visible pandemic state $E_{v}^\tau =(S^\tau +I^\tau , H^\tau , R^\tau )$, the pandemic transition history $\nabla _\tau E_v^\tau$, the collected mobility demand for the next 4 h $\{M_d^\tau ,M_d^{\tau +1},M_d^{\tau +2},M_d^{\tau +3}\}$, and the current economic loss $L^\tau$ (defined later).

The action of RL $a^\tau$ is defined with different constraints to simulate different kinds of policies. Generally, $a^\tau$ is a matrix of size $N_c \times N_c$ ($N_c$ is the number of communities), which determines the allowed mobility percentage in each pair of communities. Specifically, for city lockdown and community quarantine, we constrain $a_{i,j}^\tau = a_0^\tau$ and $a_{i,j}^\tau = a_i^\tau$, respectively.

The reward function includes two terms: an infection-spread-cost term $R_h^\tau$ and a mobility-restriction-cost term $R_m^\tau$:

9 $$\begin{aligned}&R_h^\tau = k_h \text {exp}\left( \frac{\frac{1}{K}\sum _i H_i^\tau }{H_0}\right) , \end{aligned}$$

10 $$\begin{aligned}&L_i^\tau = \sum _{t=0}^{\tau -1} \lambda ^{\tau - t} \frac{M_{d,i}^{t}- M_{p,i}^{t}}{ \overline{M_{d,i}}}, \quad R_m^\tau = \frac{1}{K} \sum _i \text {exp}\left( \frac{L_i^\tau }{L_0}\right) \frac{M_{d,i}^{\tau }- M_{p,i}^{\tau }}{\overline{M_{d,i}}}. \end{aligned}$$

Generally, the lower the health care resources (lower $H_0$), the faster $R_h^\tau$ will increase; similarly, the more vulnerable the economy (lower $L_0$), the faster $R_m^\tau$ will increase if a region is continuously restricted. The final RL reward function ${\mathcal {R}}$ is defined by:

11 $$\begin{aligned} {\mathcal {R}}\left( M_p^{\tau }, E_p^{\tau }\right) = -\left( R_m^\tau + R_h^\tau \right) . \end{aligned}$$

In this paper, we further propose a crowd-intelligent design for assessing different policies in different environments. First, we generate different environmental settings $\{(L_0, H_0)\}$, reflecting that cities have different economic vulnerabilities and health care resources. For each policy, we train multiple RL models under the same environment, record the best model, and then draw a Pareto frontier from the best results of all the environmental settings. Finally, policy assessment and comparison are conducted based on the Pareto frontier of each policy.

### Experiment settings

#### Dataset

We conduct experiments on a real-world OD dataset collected by a mobile operator in Beijing^31^. The dataset covers 544,623 residents and geographically divides Beijing into $17 \times 19$ communities. The dataset covers 24-h OD flows for the whole month of January 2019. The 1-month data are repeated 24 times to form prolonged 24-month so that we have a sufficiently long period for pandemic simulation. We summarize the details about the dataset in Table 4.

**Table 4:** The summary of the prolonged dataset.

City	Regions	Mean population	$P_{move}$	Duration
Beijing	$17\times 19$	1686	0.18	744 Days

$P_{move}$ counts the mean probability for an individual to move in 1 h.

*Privacy and ethical concerns* We have taken the following procedures to address privacy and ethical concerns. First, all of the researchers were authorized by the data provider to utilize the data for research purposes only. Second, the data is completely anonymized. Third, we store all the data in a secured off-line server.

#### Hyperparameters

Without the loss of generality, we set $\{\beta ^{s}, \beta ^{m}, \gamma ,\theta \}=\{\frac{3}{24}, \frac{0.1}{24}, \frac{0.3}{24}, and \frac{0.3}{24}\}$ for all communities at all time steps. The basic reproduction rate *R*0 is estimated as 2.1 at the initial stage of the pandemic^31^. The vaccination term $\alpha$ is 0 for most experiments and is set as $\theta =\frac{0.005}{24}$ when considering vaccination. For the reward, we mainly set $\lambda = 0.99, L_0 = 72, H_0 = 3, k_h = 1$. We also observe that policy-makers in the real world usually fail to conduct a pandemic-control policy at the time the first infection is found. Thus, in our experiments, we simulate that policy-makers begin intervention 20 days after the pandemic starts.

#### RL training

Specifically, in our experiments, we generate 5 environmental settings for each policy by adjusting *H*0 from 1 to 20 and train 16 RL models for each environmental setting. The RL models are trained on 8 V100 GPUs. The training time is approximately 8 h for each environmental setting.

#### Daily std computation

The daily std is computed by: ${\textit{std}}(\{a^t | t=0,1,\ldots ,T\})$ for city lockdown; and ${\textit{std}}(\{a^t_i | t=0,1,\ldots ,T, i=0,1,\ldots ,N\})$ for community quarantine; and ${\textit{std}}(\{a^t_{i,j} | t=0,1,\ldots ,T, i,j=0,1,\ldots ,N\})$ for route management. We set $T=90$ (4 h per control period, in a total of 90 periods in 15 days) for all policies to compare their complexity in the first 15 days of policy intervention. A large daily std means the government needs to smartly and frequently adjust restrictions, which poses high requirements on the government’s execution ability.

#### Heuristic policies

We simulate two kinds of popular real-world heuristic policies:

- *City-fixed*: Reduce all mobility in the city to a given rate. In addition to simulating the complete lockdown policy with 0% mobility, we further set the restricted mobility to 15% and 20%, since we find that they are at the boundary of successfully controlling the pandemic.
- *EP-threshold*: Similar to^31^, we define an expert policy in the community-control level: if a community’s hospitalization reaches a threshold, it will be locked down for certain days and then open for at least 1 day to satisfy the urgent mobility demands of residents.

## Data Availability

Due to privacy and ethical concerns, we are unable to provide public access to the Beijing mobility dataset. However, the dataset can be accessed upon request and the provider’s authorization. For data requests, please contact Dr. Yong Li at liyong07@tsinghua.edu.cn.

## Code availability

The RL algorithm used in this paper is available at https://github.com/AnyLeoPeace/DURLECA.
